# Parental employment during early childhood and overweight at 7-years: findings from the UK Millennium Cohort Study

**DOI:** 10.1186/s40608-015-0065-1

**Published:** 2015-09-16

**Authors:** Steven Hope, Anna Pearce, Margaret Whitehead, Catherine Law

**Affiliations:** Population, Policy and Practice Programme, UCL Institute of Child Health, 30 Guilford Street, London, WC1N 1EH UK; Department of Public Health and Policy, Institute of Psychology, Health and Society, University of Liverpool, Whelan Building, Liverpool, L69 3GB Merseyside, UK

## Abstract

**Background:**

There are increasing numbers of families with both parents (or a lone parent) employed, which may impact on the ability of families to support healthy lifestyles for their children. Some studies have linked maternal, but not paternal, employment with childhood overweight, although most have been cross-sectional or reported over short periods. We investigated the relationship between parental employment since infancy and overweight in children at 7-years. We differentiated employment by intensity (hours worked), and examined mutually adjusted associations of cumulative maternal and paternal employment with childhood overweight.

**Methods:**

Data on parental employment at 9 months, 3, 5 and 7-years were used to create cumulative measures of maternal, paternal and family employment in the UK Millennium Cohort Study (MCS). Risk ratios (RR) and 95 % confidence intervals (CI) for childhood overweight (including obesity) at age 7 were estimated according to employment, before and after adjustment for potential confounders.

**Results:**

Compared to continuous non-employment within the family since infancy, any employment of a parent was associated with lower risks of child overweight (e.g. one survey sweep in employment, adjusted RR: 0.71 [0.56–0.90]). Prolonged maternal full-time employment, however, was associated with elevated risks (four sweeps in full-time employment versus never, adjusted RR: 1.46 [1.20–1.78]). There was no equivalent association with paternal full-time employment. When limited to couple families, and adjusting for cumulative full-time employment of both parents and confounders, the risk of overweight at 7-years associated with continuous maternal full-time employment was not attenuated (adjusted RR: 1.71 [1.38–2.11]), and the association with paternal employment remained non-significant.

**Conclusions:**

Children living in workless households or where two parents are full-time employed have increased risks of overweight. These findings may imply the need for changes to enable parents to maintain healthy lifestyles for their children in the face of wider obesogenic influences.

**Electronic supplementary material:**

The online version of this article (doi:10.1186/s40608-015-0065-1) contains supplementary material, which is available to authorized users.

## Background

Levels of overweight (including obesity) have risen and remain high among children in many high-income countries, and this trend is now emerging in low and middle-income countries [1]. In England, cross-sectional data from the National Child Measurement Programme (NCMP) for the year 2012/13 suggest that 22 % of 4–5 year old and 33 % of 10–11 year old children were overweight [2]. Childhood overweight is a major public health problem, linked to increased risks of health problems in childhood and adulthood [3].

Paid employment in two-parent and lone-parent families has increased over recent decades in many high-income countries [4]. These changes mainly result from greater numbers of mothers entering the labour market [5]. Parental employment, and maternal employment in particular, has been suggested as a possible precursor of childhood overweight. It has been argued that a key mechanism through which an association between employment and childhood overweight may develop is a lack of sufficient time outside work [6]. Time constraints may result in difficulties providing a healthy lifestyle, including a balanced diet and regular mealtimes [7, 8], supporting children to get involved in physical activities [6], limiting children’s use of screen-based activities [9, 10], or walking rather than driving children to school [11]. Conversely, employment is likely to increase household income. This may have a positive impact on health-related behaviours, for example, through the purchase of healthier food and enrolment in organised physical activities, such as sports clubs [12, 13].

Time pressures are likely to be particularly apparent for parents in full-time rather than part-time employment, and where both parents (or a lone-parent) are employed. There is evidence that overweight in children is associated with intensity (hours worked) of maternal employment, with elevated risks among full-time employed mothers [10, 14–16]. Increases in maternal employment have arisen against a backdrop of relatively stable, high levels of paternal full-time employment. Thus, any relationship between maternal employment and childhood overweight occurs in the context of existing paternal full-time employment in most two-parent families. There is scant evidence that fathers’ employment is associated with childhood overweight, possibly because of little variation in employment status, and fathers generally making less of a contribution to family eating routines than mothers [8, 10]. An association between the prolonged employment of both parents in couple families and higher child BMI has been demonstrated [17]. However, the majority of evidence linking parental employment with overweight has been cross-sectional, or has not measured employment throughout the child’s life.

We aimed to investigate whether long-term exposure to parental employment from infancy to middle childhood increased the risk of overweight at 7-years. We looked at the association between duration of parental employment from 9 months to 7-years and childhood overweight in three ways. Firstly, we examined risk of overweight associated with duration exposed to *any* parental employment, regardless of hours worked, gender of any employed parent or the structure of the family. Secondly, we assessed risk associated with duration exposed to *either* maternal or paternal full-time employment. Finally, in an analysis restricted to couple families, we looked at risk of overweight according to duration of maternal *and* paternal full-time employment, mutually adjusted for each other. Analyses also took into account potential confounders that may have influenced associations between parental employment and overweight.

## Methods

### Sample

We examined data from the Millennium Cohort Study (MCS), a longitudinal study of children born in the UK between September 2000 and January 2002 which has been described elsewhere [18]. Ethical approval for each sweep of data collection was granted by NHS Research Ethics Committees (MCS1: South West MREC/01/6/19; MCS2: London MREC/03/2/022; MCS3: London 05/MRE02/46; MCS4: Yorkshire 07/MRE03/32). Parents gave informed, written consent for their participation and the participation of their child. Where parents gave consent to the participation of their child in one or more elements of a survey, inclusion required the child’s own agreement and compliance.

The first study contact took place when the cohort child was around 9-months of age (97 % of interviews took place between 8 and 11 months of age [19]). Information was collected on 72 % of those approached, providing information on 18818 infants (our analyses were restricted to 18296 singletons). Survey interviews were carried out by interviewers in the home with the main respondent (usually the mother). We used data from the first four sweeps when the children were aged 9-months, and 3, 5 and 7-years. Data were downloaded from the UK Data Archive, University of Essex in May 2010.

Our sample was limited to those families who had taken part in all four sweeps (11538). Of these, 10474 children met the criteria for inclusion in analyses. Exclusions were: 250 with missing BMI data at 7-years; a further 520 because the main respondent was someone other than the mother at one or more of the sweeps; 171 because a partner was someone other than the father at one or more sweeps; and a further 123 where there was incomplete information on the longitudinal measure of family employment. In multivariable analyses, full data were required on covariates, reducing the sample to 9827.

An additional analysis of 7728 continuous couple families excluded 2339 families because some or all of the information on fathers’ employment was missing. For these analyses the working sample was 5389, reduced to 5136 for multivariable analyses. As a sensitivity analysis, we repeated all analyses using multiple imputed data, where we imputed missing study covariates (pre-pregnancy bodysize, ethnicity, birthweight, timing of introduction to solids, highest qualification, and maternal smoking in pregnancy). Results were very similar to those for complete case analyses, and therefore only findings for complete cases are presented.

### Measures

#### Overweight

At the fourth survey sweep, interviewers weighed the children, without shoes or outdoor clothing, using Tanita HD-305 scales (Tanita UK Ltd, Middlesex, UK) and weights were recorded in kilograms to one decimal place. Heights were obtained by the Leicester Height Measure Stadiometer (Seca Ltd, Birmingham, UK) and recorded to the nearest millimetre. Childhood overweight (including obesity) was defined by the International Obesity Task Force cut-offs for BMI [20] and is age and sex standardised.

#### Parental employment

Current employment status of the mother (and the father, where applicable) was reported at the four survey sweeps available at the time (9 months, 3, 5 and 7-years). Hours of employment were also recorded, divided into part-time (≤30 h paid work per week) and full-time (>30 h). Non-employment comprised those who did not have paid work, those on long-term leave from work, and students. At each sweep, family employment variables were constructed to indicate whether at least one parent was employed, regardless of hours worked, the sex of an employed parent, or whether the family comprised one or two parents. In addition, maternal and paternal full-time employment statuses were assessed separately at each sweep. After adjusting for potential confounders selected a priori and listed below, the risk of overweight at 7-years was significantly raised only for maternal full-time employment (results not shown). Therefore, dichotomous cross-sectional variables were constructed for maternal full-time employment, with non-employment and part-time work combined as a comparison group at each sweep. The equivalent variables were also derived for full-time paternal employment.

Cross-sectional employment status variables at 9-months, 3, 5 and 7-years were summed to construct separate cumulative indicators of family employment, maternal and paternal full-time employment (ranging from 0 for no employment to 4 when employment was recorded at every sweep).

As a sensitivity test to investigate whether prolonged exposure to part-time, full-time or non-employment was associated with childhood overweight, an additional variable was constructed based on the predominant employment status of mothers over the four MCS sweeps. This variable identified whether a mother had spent at least three sweeps in a single employment status group: full-time, part-time or non-employment.

#### Potential confounders

A number of variables were identified that are known to be associated with overweight and therefore potentially confound the relationship between parental employment and childhood overweight [21]. Child characteristics included prenatal and infant feeding factors: maternal smoking in pregnancy (consuming any cigarettes throughout pregnancy or giving up during pregnancy), birthweight (converted to z-scores, adjusting for sex and gestational age), pre-term birth (before 37-weeks gestation), breastfeeding duration and timing of introduction to solids. Characteristics of mothers included: highest educational qualification, ethnicity, age at cohort child’s birth, and pre-pregnancy bodysize (self-reported pre-pregnancy weight and current height, with a BMI ≥ 25 kg/m^2^ considered to be overweight). Birthweight is presented as a categorical measure (Table 1), although it was entered into models as a continuous variable. Mothers’ ethnicity is presented as dichotomous (White, Non-White) but in the models the variable was entered as “British-white”, “White-other”, “Mixed”, “Indian”, “Pakistani or Bangladeshi”, “Black or Black British” and “Other”.

**Table 1:** Weighted percentages for prevalence of potential covariates and associations with overweight at 7-years

	Percent (*n*) Total	Percent (*n*) Not overweight at 7 years	Percent (*n*) Overweight at 7 years
		Chi-square test	
Birthweight			
Normal/high birthweight (≥2500 g)	94.2 (9784)	93.9 (7775)	95.3 (2009)
Low birthweight (<2500 g)	5.8 (596)	6.1 (499)	4.7 (97)
		*p* < 0.05	
Pre-term birth			
Normal (≥37 weeks)	93.1 (9708)	93.2 (7738)	93.0 (1970)
Pre-term (<37 weeks)	6.9 (695)	6.8 (552)	7.1 (143)
		*p* = 0.77	
Maternal age at child’s birth			
14–19 years	7.2 (647)	7.2 (515)	7.1 (132)
20–29 years	44.6 (4575)	44.7 (3634)	44.5 (941)
30–39 years	45.9 (4994)	46.0 (3996)	45.7 (998)
40+ years	2.2 (258)	2.1 (200)	2.8 (58)
		*p* = 0.49	
Maternal ethnicity			
White	88.0 (9057)	88.4 (7234)	86.3 (1823)
Non-white	12.0 (1393)	11.6 (1091)	13.7 (302)
		*p* < 0.05	
Maternal pre-pregnancy bodysize			
Normal / underweight	71.0 (6964)	75.0 (5871)	54.7 (1093)
Overweight	29.0 (2988)	25.0 (2071)	45.3 (917)
		*p* < 0.001	
Family structure (9-months–7-years)			
Continuous couple families	72.1 (7728)	73.1 (6227)	67.9 (1501)
Couple families for 1–3 sweeps	20.9 (2042)	20.2 (1594)	23.4 (448)
Continuous lone-parent families	7.1 (704)	6.7 (524)	8.8 (180)
		*p* < 0.001	
Maternal highest qualification (9-months)^a^			
Degree	17.9 (2028)	19.1 (1729)	12.8 (299)
Diploma	9.4 (1026)	9.5 (820)	9.1 (206)
A/AS/S Levels	9.8 (1088)	10.1 (887)	8.8 (201)
O Levels/GCSEs A-C	35.7 (3592)	35.2 (2797)	37.7 (795)
O Levels/GCSEs D-G	10.9 (1057)	10.5 (817)	12.6 (240)
Other academic qualification	2.0 (218)	1.8 (171)	2.5 (47)
None of these qualifications	14.3 (1456)	13.8 (1117)	16.6 (339)
		*p* < 0.001	
Breastfeeding duration			
Never	31.2 (3161)	30.4 (2434)	34.6 (727)
Up to 4 months	40.8 (4407)	40.7 (3515)	41.0 (892)
Greater than 4 months	28.0 (2906)	28.9 (2396)	24.4 (510)
		*p* < 0.001	
Introduced to solids before 4-months of age			
No	64.0 (6753)	64.9 (5462)	60.4 (1291)
Yes	36.0 (3719)	35.1 (2881)	39.6 (838)
		*p* < 0.001	
Maternal smoking during pregnancy			
No	65.6 (7006)	66.6 (5676)	61.7 (1330)
Yes	34.4 (3439)	33.4 (2645)	38.3 (794)
		*p* < 0.001	
Number of children in household at first sweep (9-months)			
1	42.0 (4365)	42.3 (3484)	40.8 (881)
2	36.6 (3755)	36.7 (2994)	36.3 (761)
3	14.5 (1592)	14.4 (1275)	15.2 (317)
4+	6.9 (762)	6.7 (592)	7.7 (170)
		*p* = 0.29	

^a^Maternal highest academic qualification achieved: Ranging from highest (degree) to lowest (General Certificate of Education (GCE) Ordinary (O) Levels / General Certificate of Secondary Education (GCSE) grades D-G), plus categories for those with no qualifications or others not listed

Missing data for covariates: birthweight 94; gestation 71; maternal age at birth of child 0; maternal ethnicity 24; pre-pregnancy weight 522; family structure 0; maternal education 9; breastfeeding 0; solids 2; smoking 29; children in household 9-months 0

Family structure was identified a priori as a potential moderator of the relationship between parental employment and child overweight and examined as an interaction term, with family structure defined longitudinally over the four sweeps (continuous couple families, couple families for 1–3 sweeps, or continuous lone-parent families).

### Statistical analysis

Univariable analyses were undertaken to explore distributions of variables and associations with overweight at 7-years. Only those covariates associated with overweight were included in subsequent multivariable models. When multivariable analyses were repeated including the non-significant covariates, findings were similar to those reported (results not shown).

Multivariable Poisson regression was used to estimate risk ratios (RR) and 95 % confidence intervals (CIs) for child overweight at 7-years, modelling the effects of cumulative family employment, and cumulative full-time maternal and paternal employment separately. Poisson regression provides reliable estimates of risk ratios when an outcome is common [22, 23]. The final series of models were carried out in families where there had been two parents in all sweeps, providing an opportunity to estimate the unique contribution of each parents’ cumulative full-time employment on overweight at 7-years. Continuous couples were the largest family structure group and there were insufficient numbers to examine lone-parent families longitudinally. An interaction between family structure and cumulative maternal full-time employment was statistically significant (*p* < 0.01), indicating differential associations with employment according to whether or not there had been lone-parenthood or family disruption. The effects of maternal and paternal full-time employment were tested separately for couple families, and then in combination by mutually adjusting for maternal and paternal cumulative full-time employment. The unadjusted models described were repeated with adjustment for the potential confounders. All multivariable analyses were carried out using complete samples so that RRs could be directly compared before and after adjustment.

Analyses were conducted in Stata/SE 12.1 (Stata Corporation, TX). Weighted percentages, univariable and adjusted analyses were estimated using ‘svy’ commands to allow for the clustered sampling design and attrition.

## Results

### Descriptive statistics

Twenty percent (2161) of children in the sample were overweight or obese at 7-years, of which 6 % (629) were classified as obese using IOTF cut-offs. Most of the potential confounding variables were associated with weight status at 7-years (Table 1), with greater risks of overweight associated with pre-pregnancy and pregnancy factors, early feeding practices, and lower educational qualification levels. Children of low birthweight had a lower risk of overweight at 7-years.

Maternal employment increased from less than half the sample (47 %) when the cohort child was 9-months of age to almost two-thirds at 7-years (63 %). In contrast, levels of paternal employment were relatively stable, above 90 % at each of the four survey sweeps, with 87 % of fathers continuously-employed throughout. There was no evidence of a cross-sectional association between maternal or paternal employment *per se* (not differentiating hours worked) at any sweep and childhood overweight at 7-years.

### Family employment

Over the duration of the study, few families reported that they never had a parent in employment (7 %), and the majority always had at least one working parent at every sweep (74 %). Table 2 shows the relationship between any employment within the family (couple or lone-parent) from 9-months to 7-years and overweight at 7-years, with lower risks among children who had at least one employed parent for one or more sweeps since infancy, compared to a baseline of continuous non-employment within the family, before and after adjustment for potential confounders.

**Table 2:** Weighted percentages for prevalence and risk ratios (95 % CIs) for overweight at 7-years by cumulative family employment, unadjusted and adjusted for confounders (*n* = 9827)

	Percent (*n*) overweight	Unadjusted RR	+ Confounders^b^
*Family employment*			
None^a^	28.2 (147)	1	1
One sweep	19.7 (99)	0.70 (0.55–0.89)	0.71 (0.56–0.90)
Two sweeps	21.8 (98)	0.77 (0.60–0.99)	0.79 (0.62–1.00)
Three sweeps	22.3 (188)	0.79 (0.64–0.98)	0.82 (0.66–1.01)
Four sweeps	18.7 (1454)	0.66 (0.55–0.79)	0.75 (0.62–0.89)

^a^Baseline group: no sweeps at which any employment was reported by a parent

^b^Confounders: maternal ethnicity, highest qualification, pre-pregnancy bodysize, smoking in pregnancy, birthweight, duration breastfeeding, solids at 4-months

### Maternal and paternal full-time employment

Maternal employment was largely part-time (≤30 h), and almost three-quarters of mothers had not been employed full-time at any of the sweeps up to 7-years (74 %). Cumulative maternal full-time employment was associated with overweight at 7-years, particularly when recorded over two or more sweeps (Table 3). In multivariable analyses, adjustment for confounders did not attenuate the relationship between maternal cumulative full-time employment and child overweight. As a sensitivity analysis, we differentiated full-time, part-time or non-employment for three or more sweeps. The earlier results were confirmed: children of predominantly full-time employed mothers were at increased risk of overweight at 7-years, compared to those who were either predominantly non-employed or part-time employed, both before and after adjustment (results in Additional file 1: Table S1).

**Table 3:** Weighted percentages for prevalence and risk ratios (95 % CIs) for overweight at 7-years by cumulative maternal full-time employment, unadjusted and adjusted for confounders (*n* = 9827)

	Percent (*n*) overweight	Unadjusted RR	+ Confounders^b^
*Maternal full-time employment*			
None^a^	19.3 (1365)	1	1
One sweep	17.4 (217)	0.90 (0.77–1.06)	0.91 (0.78–1.07)
Two sweeps	23.1 (142)	1.20 (1.01–1.43)	1.21 (1.01–1.44)
Three sweeps	23.6 (102)	1.23 (0.99–1.52)	1.26 (1.01–1.58)
Four sweeps	26.8 (160)	1.39 (1.15–1.68)	1.46 (1.20–1.78)

^a^Baseline group: no sweeps at which full-time employment was reported by the mother

^b^Confounders: maternal ethnicity, highest qualification, pre-pregnancy bodysize, smoking in pregnancy, birthweight, duration breastfeeding, solids at 4-months

Paternal employment was largely full-time (>30 h paid work per week) at all sweeps (79 %). Analysis of cumulative full-time employment indicated no association between duration of full-time paternal employment and child overweight at 7-years (as shown in Table 4).

**Table 4:** Weighted percentages for prevalence and risks ratios (95 % CIs) for overweight at 7-years by cumulative maternal and paternal full-time employment: continuous couple families only (*n* = 5136)

	Percent (*n*) overweight	Unadjusted RR	Mutually adjusted cumulative maternal and paternal employment	Mutually adjusted + Confounders^b^
		**A**	**B**	**C**
*Maternal full-time employment*				
None^a^	17.3 (635)	1	1	1
One sweep	13.5 (98)	0.78 (0.63–0.97)	0.78 (0.63–0.97)	0.79 (0.64–0.97)
Two sweeps	21.5 (70)	1.24 (0.94–1.64)	1.25 (0.94–1.65)	1.29 (0.97–1.71)
Three sweeps	24.0 (60)	1.39 (1.02–1.89)	1.39 (1.02–1.89)	1.44 (1.04–1.99)
Four sweeps	29.0 (106)	1.68 (1.36–2.07)	1.69 (1.37–2.08)	1.71 (1.38–2.11)
*Paternal full-time employment*				
None^a^	17.8 (36)	1	1	1
One sweep	18.4 (28)	1.03 (0.60–1.79)	1.04 (0.60–1.80)	1.06 (0.61–1.84)
Two sweeps	11.8 (32)	0.66 (0.39–1.13)	0.65 (0.38–1.11)	0.70 (0.41–1.18)
Three sweeps	20.4 (118)	1.15 (0.79–1.67)	1.14 (0.78–1.65)	1.18 (0.82–1.70)
Four sweeps	18.1 (755)	1.01 (0.71–1.44)	1.01 (0.71–1.45)	1.11 (0.79–1.56)

^a^Baseline group: no sweeps at which full-time employment was reported by the parent

^b^Confounders: maternal ethnicity, highest qualification, pre-pregnancy bodysize, smoking in pregnancy, birthweight, duration breastfeeding, solids at 4-months

### Mutually adjusted maternal and paternal full-time employment

The relationships between both maternal and paternal cumulative full-time employment and overweight were investigated in continuous couple families (Table 4). Unadjusted risks for overweight at 7-years were calculated separately for maternal and paternal employment (Column A). Elevated risks associated with exposure to maternal full-time employment over multiple sweeps were again observed, with risks associated with long-term full-time employment appearing even greater when restricted to mothers in couple families. Risks associated with cumulative maternal full-time employment persisted after adjustment for cumulative paternal full-time employment (Column B), and confounders (Column C). There remained no association between paternal full-time employment and overweight for any of these analyses. Similar results to those in Column C for maternal and paternal employment were obtained when highest paternal qualification and both maternal and paternal highest qualifications were included in the model adjusting for confounders (results not shown).

## Discussion

Using longitudinal data on parental employment, we found that children aged 7-years were at a reduced risk of overweight (including obesity), compared to workless households, if they had at least one parent in employment for one or more sweeps during the period from early infancy to middle childhood. This suggests that extended periods in workless households may lead to an elevated risk of childhood overweight. Conversely, the risk of overweight was also elevated if the mother reported prolonged full-time employment during this period. There was no equivalent association between childhood overweight and part-time maternal employment or paternal employment (regardless of hours worked), and risks were not attenuated by adjustment for potential confounders. Further analyses restricted to couple families with employment data for both mothers and fathers showed that risks associated with maternal employment remained after adjustment for paternal employment.

Our findings support a body of research evidence that has shown hours of maternal employment to be associated with overweight in childhood, with elevated risks limited to, or stronger, for mothers in full-time employment [24, 25]. The absence of an association with paternal employment is also consistent with existing evidence [14]. Unlike many earlier studies, we examined the combined influence of paternal and maternal employment in couple families and showed that the risks associated with prolonged maternal full-time employment were independent of paternal employment status. Furthermore, associations were notably larger in couple families in comparison with all family types combined. Our findings may be the consequence of the predominance (and lack of variation) of full-time employment among fathers [17] or the central role mothers continue to play in the domestic routines of a family [16]. This suggests that one potential mechanism through which full-time maternal employment is associated with childhood overweight may be through less health promoting behaviours associated with parental time constraints [6], within the context of environmental influences outside of the immediate home and family which are often obesogenic.

### Strengths and limitations

A major strength of these analyses is the use of the UK Millennium Cohort Study, a large, nationally-representative contemporary cohort. Height and weight of the study children were recorded objectively by trained interviewers. The extensive information collected in the MCS interviews allowed us to account for a range of early life characteristics and to demonstrate potentially important differences in relationships between maternal and paternal employment status and childhood overweight, including hours worked. The longitudinal aspect of the MCS allowed us to investigate parental employment over the first 7 years of a child’s life, which is particularly useful given that overweight will develop gradually. We did not investigate possible causal mechanisms, such as the extent to which an association between employment and overweight was due to lifestyle factors including diet, sedentary behaviour and physical activity, since measures of these in the MCS are relatively crude and some have not been collected consistently across sweeps. Also, we did not include time-varying confounders, or BMI of the child at intermediate sweeps, given our aim was to investigate the cumulative relationship between parental employment and overweight in middle childhood rather than testing mechanisms or associations with earlier BMI [10, 26].

Despite the strengths of cohort data, attrition is always a concern in longitudinal research. There was no evidence that children who were overweight or obese at 7-years were less likely to have taken part in all four sweeps than those within the normal weight range (Overweight: 85 % vs. Normal weight: 84 %, *p* > 0.05), nor for those who were overweight at 3 or 5-years. However, parental employment status was associated with non-response. Children living in families with no employed parent were less likely to have taken part in all four sweeps, and this held for non-employment reported at any of the four sweeps (for example, 73 % of children without an employed parent at 7-years had taken part in all sweeps vs. 87 % of those with an employed parent, *p* < 0.001). The same pattern of attrition was also found for maternal and paternal non-employment. Therefore, the possibility that results may have been subject to bias cannot be dismissed, although we used response weights to account for attrition up to the 7-year survey, and findings from a sensitivity analysis with imputed data were similar to those reported.

When investigating the relationship between full-time employment and child overweight, the comparison group adopted was non-employment combined with part-time employment. This comparison group is heterogeneous, comprising parents with potentially very different working and non-working lives and access to financial resources. Those women with part-time employment were likely to be particularly varied, sharing characteristics with full-time employed women (in receipt of a salary) and those who were non-employed (greater opportunity for time with family). Nevertheless, both non-employed and part-time employed women are likely to share a key characteristic of interest in this analysis; namely, increased time to allocate to their family for food preparation and involvement in activities with the child, in comparison to a full-time employed parent. Non-employment and part-time employment have been combined in previous studies [27], and there is evidence, both from our preliminary analyses and in earlier research, that risks of childhood overweight do not differ between the children of women who are employed part-time and those who are non-employed [14]. We attempted to address the issue of heterogeneity of the comparison group in two ways. First, we controlled for potential confounding socio-demographic and biological factors in multivariable modelling, both for preliminary analyses of cross-sectional employment status and overweight at 7-years (there was no significant difference between non-employment and part-time employment at any sweep after adjustment for confounders), and in models testing cumulative full-time employment. Second, we carried out a sensitivity analysis in a subsample of the mothers, comparing child overweight for those who had occupied a single employment status in three or more of the four survey sweeps (non-employment, part-time and full-time employment). The findings supported the results for cumulative full-time employment, with elevated levels of overweight in children of full-time employed mothers.

A final potential limitation is that employment status was based on that currently reported at each of the four sweeps, and so will have overlooked fluctuations in employment status occurring between sweeps. However, these data provide insights into the working lives of parents during early childhood, and will be less prone to the recall bias likely to result from retrospective employment histories. Nonetheless, a more detailed analysis of the characteristics and context of employment was beyond the scope of these analyses.

### Implications for research

Quantitative research using large datasets can identify associations between potentially important explanatory factors, but other research designs, such as qualitative studies, may be better able to investigate underlying mechanisms linking employment with childhood overweight. Modern families’ lives are complex, and quantitative studies can only provide a crude summary of this reality. Nevertheless, it will be important to continue to investigate associations between parental employment and overweight as the MCS children move into adolescence, particularly since there is evidence that relationships may change as children gain independence from family supervision [28].

## Conclusions

Government policies in high-income countries over a number of years have promoted the uptake of paid work among parents as a route out of poverty. Our findings indicate that living in a household where at least one parent was employed was associated with a lower rate of childhood overweight. However, maternal full-time employment was associated with increased risks of overweight and may also have weakened the otherwise protective effects of socio-economic advantage. Against a backdrop of widespread paternal full-time employment in the MCS, our results indicate that risks for childhood overweight were elevated when both parents in couple families were employed full-time, implying the need for policies to help them to maintain a healthy lifestyle for their children growing up in an environment outside of the family environment which is often obesogenic. Such policies might include making part-time and flexible working more practical and realistic for the mothers and fathers of young children. Additionally, the cumulative nature of the association between full-time employment during the period from infancy through to middle childhood and overweight suggests the need to consider factors more broadly related to employment during this period. Studies have shown that childcare settings may play an important role in the development of healthy weight, particularly through the provision of healthy diets in pre-school years, with informal childcare linked to increased risks of overweight [29–31]. Such findings, taken together with those reported here, suggest the importance of provision of healthy meals and adequate physical activity in formal and informal childcare in preschool and the early school years, and active transport to school.

## Additional file

Additional file 1: Table S1. Weighted percentages for prevalence and risk ratios (95 % CIs) for overweight at 7-years by predominant maternal employment status, unadjusted and adjusted for confounders (*n* = 7675). (DOC 28 kb)
