# Supplementary material for: Parental employment during early childhood and overweight at 7-years: findings from the UK Millennium Cohort Study
Source: BMC Obes. 2015 Sep 16;2:33. doi: 10.1186/s40608-015-0065-1 (PMC4571056; doi:10.1186/s40608-015-0065-1)
Supplement: Additional file 1: Table S1. — Weighted percentages for prevalence and risk ratios (95 % CIs) for overweight at 7-years by predominant maternal employment status, unadjusted and adjusted for confounders (n = 7675). (DOC 28 kb) [file 40608_2015_65_MOESM1_ESM.doc]

**Table S1: Weighted percentages for prevalence and risk ratios (95% CIs) for overweight at 7-years by predominant maternal employment status^†^, unadjusted and adjusted for confounders (n=7675)**

|  | **Percent (N) overweight** | **Unadjusted RR** | **+ Confounders**^§^ |
| --- | --- | --- | --- |
| *Predominant employment status* |  |  |  |
| Non-employment^‡^ | 20.0 (702) | 1 | 1 |
| Part-time employment | 18.1 (586) | 0.90 (0.81-1.01) | 0.99 (0.89-1.11) |
| Full-time employment | 25.4 (262) | 1.27 (1.10-1.46) | 1.36 (1.17-1.58) |

†Three or more sweeps recorded in a single employment status group

‡Baseline group: three of more sweeps where no employment was reported by the mother

§Confounders: maternal ethnicity, highest qualification, pre-pregnancy bodysize, smoking in pregnancy, birthweight, duration breastfeeding, solids at 4-months
